# Psychometric Properties and Factor Structure of the Slovak Version of the Copenhagen Burnout Inventory

**DOI:** 10.3390/ijerph191912586

**Published:** 2022-10-02

**Authors:** Karol Kováč, Júlia Halamová

**Affiliations:** 1 Institute of Applied Psychology, Faculty of Social and Economic Sciences, Comenius University in Bratislava, 814 99 Bratislava, Slovakia; 2 Institute of Stress, 917 24 Trnava, Slovakia

**Keywords:** burnout, Copenhagen Burnout Inventory, factor analysis, reliability, validity

## Abstract

Background: Burnout is clinically significant because it has adverse consequences in the form of mental and physical illnesses and economic losses. The goal of the research study was to translate the Copenhagen Burnout Inventory (CBI) into Slovak and then assess its factor structure, reliability, validity, and psychometric properties. Methods: We collected two convenience samples: one for the psychometric properties analysis and factor analysis, which consisted of 4789 participants, and the other for the validity analysis, which consisted of 458 participants. Data were collected online. The participants filled out the following questionnaires: Copenhagen Burnout Inventory (CBI), Oldenburg Burnout Inventory (OLBI), Professional Quality of Life Scale (PROQOL), and Secondary Traumatic Stress Scale (STSS). Results: The CBI had very good reliability in both samples, calculated using Cronbach alpha coefficients (between 0.839 and 0.908). In terms of CBI validity, all the correlation coefficients between the scale and subscales were between moderately high and high and ranged from 0.505 to 0.859. Except for two items, CBI7 and CBI10, all the items fall into the original factors of the scale. Conclusions: The Slovak version of the Copenhagen Burnout Inventory is a statistically sound instrument with high coefficients of reliability as well as validity and has an acceptable factor structure.

## 1. Introduction

Burnout first came to the attention of the scientific community when introduced by Freudenberger (1974) [1] and Maslach (1976) [2]. Since then, burnout has become a well-recognized and widespread concept internationally. Burnout can be referred to as “a state of mental exhaustion resulting from chronic stress in the working situation” [3]. Burnout is clinically significant because it has adverse consequences in the form of mental and physical illnesses and economic losses. The International Classification of Diseases (ICD-11) [4] depicts burnout as reduced professional self-efficacy, energy exhaustion, and mental negativism and distancing related to work. However, burnout is still not included in the latest edition of the Diagnostic and Statistical Manual of Mental Disorders (DSM-V) [5]. Some scholars assume it will be included in the sixth revision of the DSM-VI, while others consider it to be depression-based and therefore not a disease in its own right [6].

The negative impact of providing help is often discussed using a variety of terms, such as secondary traumatic stress, client-related burnout, second victim traumatization, vicarious traumatization, or compassion fatigue. Some authors have already tried to differentiate between these constructs (e.g., Thomas and Wilson, 2004) [7], but so far, there is no evidence that these constructs are in fact conceptually different [8,9].

There are several instruments for measuring burnout. For several decades, the Maslach Burnout Inventory (MBI) [10,11] had the monopoly in scientific studies of burnout across the world [12]. Worley et al. (2008) [13] conducted a meta-analysis of studies testing the MBI’s factor analysis and confirmed it had a three-factor structure. However, there were numerous criticisms of the MBI regarding methodological issues, such as the lack of a total burnout score and the fact that it had both positively and negatively worded items and was not a cross-culturally sensitive tool (e.g., Kristensen et al., 2005) [14]. In response to these criticisms, other burnout instruments were developed, such as the Copenhagen Burnout Inventory (CBI) [14] or the Oldenburg Burnout Inventory (OLBI) [15]. Additionally, a couple of burnout instruments were developed in relation to specific areas, such as the School Burnout Inventory [16] or Parental Burnout Inventory [17]. The newest scale is The Burnout Assessment Tool (BAT) [18], which was developed using a combination of a theoretical and an empirical approach on the basis of a number of in-depth interviews with professionals and analysis of over a dozen burnout questionnaires that included more than 300 items. Of these newly developed burnout tools, the CBI is cited the most (based on a google scholar search by the authors in January 2022).

## 2. Copenhagen Burnout Inventory (CBI)

The CBI consists of three separate subscales to measure burnout [14]: 1/personal burnout (6 items for example “How often do you think: ‘I can’t take it anymore’?”), measuring the level of physical and psychological burnout reported by a person irrespective of their workload, 2/work-related burnout (7 items for example “Are you exhausted in the morning at the thought of another day at work?”), measuring the level of burnout related to the workload, and 3/client-related burnout (6 items for example “Do you sometimes wonder how long you will be able to continue working with clients?”), measuring the level of physical and psychological burnout reported by persons working with clients. The scale was developed to enable cross-people comparisons regardless of occupational status [14]. The authors focus on people attributing warning signs to their work and the extent to which people link their fatigue with working with other people. In the CBI the term client can be substituted by an appropriate term for the specific target group. All the CBI items are positively worded. The CBI has 19 items altogether, and the answer format uses a 5-point scale (1—never/almost never/to a very low degree; 5—always/to a very high degree). Higher scores point to higher burnout, scores < 50 reflect no or a low level of burnout, 50–74 moderate burnout levels, and scores > 75 mean high or a severe level of burnout [19].

Cronbach alpha coefficients for the CBI ranged between 0.84 and 0.91 for personal burnout, 0.84 and 0.90 for work-related burnout, and 0.84 and 0.92 for client related burnout [20,21].

Kristensen et al. (2005) [14] recommended translating the CBI into different languages and testing it in different cultures. Subsequently, the CBI has been translated and used in research studies in the following languages: Arabic [22], Chinese [23], Danish [14], English [21], Greek [20], Italian [24], Japanese [25], Korean [26], Malay [27], Persian [28], Portuguese [29], Serbian [30], Slovenian [31], Spanish [32], Thai [33], and many others. Most have been published quite recently, probably due to the onset of the COVID-19 pandemic. However, the CBI has not yet been translated into Slovak or tested in Slovakia.

## 3. Aim of the Study

The goal of the research study was to translate the Copenhagen Burnout Inventory (CBI) [14] into Slovak and subsequently assess its psychometric properties, reliability, validity, and factor structure.

## 4. Research Methods

### 4.1. Instruments

Apart from the Copenhagen Burnout Inventory (CBI) [14], for the analysis of the CBI we used the Oldenburg Burnout Inventory (OLBI) [15], Professional Quality of Life Scale (PROQOL) [34], and Secondary Traumatic Stress Scale (STSS) [35], as some authors use the constructs compassion fatigue and secondary traumatic stress interchangeably with burnout, second victim traumatization, or vicarious traumatization [8,9]. Comparably, compassion satisfaction would be the opposite construct to compassion fatigue as well as burnout.

### 4.2. Copenhagen Burnout Inventory 

The translation from English to Slovak language was done by an authorized professional. We double-checked the correctness of the translation by translating back into the original Slovak version and comparing it and again checking for the correct meaning. After the translation we did a pre-test on a sample of 50 participants. The final version of the Slovak translation of the CBI items is as follows:

Personal burnout/Osobné vyhorenie
  CBI1 How often do you feel tired?/Ako často cítite únavu?
  CBI2 How often are you physically exhausted?/Ako často cítite fyzickú vyčerpanosť?
  CBI3 How often are you emotionally exhausted?/Ako často cítite emocionálnu vyčerpanosť?
  CBI4 How often do you think: “I can’t take it anymore”?/Ako často si pomyslíte: “Už to nezvládam”?
  CBI5 How often do you feel worn out?/Ako často sa cítite opotrebovane?
  CBI6 How often do you feel weak and susceptible to illness?/Ako často sa cítite slabo a náchylne na ochorenia?

Work-related burnout/Pracovné vyhorenie
  CBI7 Do you feel worn out at the end of the working day?/Cítite sa na konci pracovného dňa uťahane?
  CBI8 Are you exhausted in the morning at the thought of another day at work?/Keď si ráno pomyslíte na ďalší deň v práci, cítite sa vyčerpane?
  CBI9 Do you feel that every working hour is tiring for you?/Máte pocit, že je pre vás každá hodina v práci vyčerpávajúca?
  CBI10 Do you have enough energy for family and friends during leisure time?/Máte vo svojom voľnom čase dostatok energie pre rodinu a priateľov?
  CBI11 Is your work emotionally exhausting?/Je vaša práca emocionálne vyčerpávajúca?
  CBI12 Does your work frustrate you?/Frustruje vás vaša práca?
  CBI13 Do you feel burnt out because of your work?/Cítite sa vyhorene zo svojej práce?

Client-related burnout/Klientské vyhorenie
  CBI14 Do you find it hard to work with clients?/Je pre vás ťažké pracovať s ľuďmi?
  CBI15 Does it drain your energy to work with clients?/Práca s ľuďmi vás energeticky vyčerpáva?
  CBI16 Do you find it frustrating to work with clients?/Cítite, že práca s ľuďmi vás frustruje?
  CBI17 Do you feel that you give more than you get back when you work with clients?/Máte pocit, že pri práci s ľuďmi oveľa viac dávate ako dostávate?
  CBI18 Are you tired of working with clients?/Cítite sa z práce s ľuďmi unavene?
  CBI19 Do you sometimes wonder how long you will be able to continue working with clients?/Uvažujete niekedy nad tým, ako dlho ešte dokážete pracovať s ľuďmi?

### 4.3. Oldenburg Burnout Inventory 

The Oldenburg Burnout Inventory (OLBI) was developed for the general population (OLBI) [15]. The scale consists of 16 items with Likert type scoring from 1 (strongly agree) to 4 (strongly disagree). The OLBI has two subscales: exhaustion and disengagement. The OLBI has been backtranslated into Slovak [36]. In our sample, the Cronbach alpha coefficients were 0.806 for exhaustion and 0.705 for disengagement.

### 4.4. Professional Quality of Life Scale 

As compassion fatigue is semantically close to burnout, we also used the PROQOL to validate the CBI by measuring levels of compassion fatigue and its counterpart compassion satisfaction. The PROQOL [9] has 30 items and three subscales: positive (compassion satisfaction, CS) and negative (compassion fatigue, which consists of secondary traumatic stress, STS, and burnout, BO). Each subscale consists of 10 items and answers are given on a 5-point scale (1 for never and 5 for very often) related to helping experiences in the last 30 days. The scale was translated into Slovak and showed good psychometric properties [37,38]. In our sample, the Cronbach alpha coefficients were 0.809 for burnout, 0.887 for compassion satisfaction, and 0.838 for compassion fatigue.

### 4.5. Secondary Traumatic Stress Scale 

The Secondary Traumatic Stress Scale (STSS) [35] captures secondary traumatic stress and consists of 17 items rated on a 5-point Likert-type scale (1 = never, up to 5 = very often) and three subscales: intrusion, avoidance, and arousal. The scale measures the frequency of symptoms related to traumatic event exposure in a helping relationship in the past seven days. It is possible to calculate the total score of the scale as well as the scores for each subscale. The STSS was backtranslated into Slovak [36]. In our sample, the Cronbach alpha coefficients were 0.845 for avoidance, 0.852 for arousal, and 0.812 for intrusion.

## 5. The Research Sample

We collected two convenience samples: one for the psychometric properties analysis and factor analysis, which consisted of 4789 participants, and the other for the validity analysis, which consisted of 458 participants.

Our first convenience sample, used for the psychometric properties analysis and factor analysis, consisted of 4789 respondents: 49% were men and 51% women. Age of respondents ranged from 18 years to 64 years. Four percent of participants were aged between 18 and 24 years, 35% between 25 and 34, 38% between 35 and 44, 21% between 45 and 54, and 2% between 55 and 64. Data were collected online. Due to the GDPR, there were no other sociodemographic data gathered.

Our second convenience sample, used for the validity analysis, consisted of 458 respondents: 6.8% were men and 92.8% women. Age of respondents ranged from 18 years to 76 years. The age breakdown is as follows: 5.5% of participants were aged between 18 and 24 years, 20.3% between 25 and 34, 25.8% between 35 and 44, 31.2% between 45 and 54, 14% between 55 and 64, and 3.2% between 65 and 76. Data were collected online.

All participants in both samples completed an online informed consent form. Data were collected in accordance with the ethical standards of the 1964 Helsinki Declaration and its later amendments or comparable ethical standards. The study’s protocol was approved by the ethical committee of the Faculty of Social and Economic Sciences at Comenius University in Bratislava, Slovakia.

## 6. Data Analysis

For the data analysis, we used IBM SPSS program version 26 (IBM Corp., Armonk, NY, USA).

## 7. Results

### 7.1. Psychometric Analysis of the CBI Items

In Table 1 we report the descriptive statistical analysis of the CBI for sample one and in Table 2 for sample two. We also calculated a one-sample Kolmogorov-Smirnov test to test the normality of the items and all the items have nonnormal distribution.

### 7.2. CBI Reliability Analysis

In order to test the CBI reliability, we calculated the Cronbach alpha coefficients: These were personal burnout: 0.845 in sample one and 0.908 in sample two, work-related burnout: 0.839 in sample one and 0.869 in sample two, and client-related burnout: 0.850 in sample one and 0.879 in sample two. In addition, we also calculated McDonald´s omega and the results were as follows: personal burnout: 0.847 in sample one and 0.910 in sample two, work-related burnout: 0.858 in sample one and 0.876 in sample two, and client-related burnout: 0.851 in sample one and 0.880 in sample two.

### 7.3. CBI Validity Analysis

In order to test the CBI validity, we calculated the Spearman correlations between the selected scales and the OLBI exhaustion and disengagement subscales [15], the PROQOL compassion fatigue, burnout, and compassion Satisfaction [9], and the STSS intrusion, avoidance, and arousal [35]. All correlations were high and ranged from 0.505 to 0.859 regardless of the direction of the correlation; see Table 3. The convergent analysis of the CBI subscales (personal burnout, work-related burnout, and client-related burnout) was performed by calculating correlations with burnout PROQOL, compassion fatigue PROQOL, disengagement OLBI, exhaustion OLBI, intrusion STSS, and arousal STSS. The discriminant analysis of the CBI subscales was provided by calculating correlations with compassion satisfaction PROQOL. As we used ordinal scales, we calculated Spearman correlations. However, due to metanalyses reasons, we also added calculations of Pearson correlation; see Table 4.

### 7.4. Factor Analysis

The exploratory factor analysis was calculated as there has been no research on factor analysis in its Slovak version yet. The factor analysis was calculated by means of principal axis factoring with rotation direct oblimin as the items were nonnormal and the factors strongly correlated. The results of the factor analysis are given in Table 5. The KMO was 0.937, so the sampling adequacy is good, and the results of the Bartlett’s test was χ^2^(171) = 32,016,914, *p* < 0.001.

CBI client-related burnout is compact and all the original items in Kristensen et al. (2005) [14] fall into the factor in the Slovak sample. However, two items originally belonging to the CBI work-related burnout fall into personal burnout, namely, CBI7 “Do you feel worn out at the end of the working day?” and CBI10 “Do you have enough energy for family and friends during leisure time?” We suggest a different formulation of the items to fit the originally intended subscales. CBI7 “Do you feel worn out at the end of the working day?/Cítite sa na konci pracovného dňa uťahane?” could be changed to remain in work-related burnout to “Do you feel worn out leaving your workplace?/Cítite sa uťahane keď odchádzate z práce?” CBI10 “Do you have enough energy for family and friends during leisure time?/Máte vo svojom voľnom čase dostatok energie pre rodinu a priateľov?” could be changed to remain in work-related burnout to “After work do you have enough energy for leisure time activities?/Po práci mate dosť energie na voľnočasové aktivity?”

## 8. Discussion

In this research study, the goal was to translate the Copenhagen Burnout Inventory (CBI) [14] into Slovak and assess its factor structure, reliability, validity, and psychometric properties. All the CBI items are skewed in the sense that most of the participants’ answers are linked to smaller burnout scores. Most of the CBI items indicated skewness and kurtosis values, which suggests the items have a nonnormal distribution. The Cronbach reliability coefficients were high in all CBI subscales as well as in both samples, which means that the items of the subscales were closely related as a group. Our results correspond with the reliability reported in previous studies [14,20,21]. The Spearman correlation coefficients are moderate; all are more than 0.505. As expected, only the correlations with the PROQOL compassion satisfaction [9] are negative because the constructs behind the scales run counter to each other: compassion satisfaction refers to the joy of helping others and being devoted and efficient in work [9]. Moderately high positive correlations between CBI burnout and PROQOL compassion fatigue [9] and STSS secondary traumatic stress [35] support academics who think these constructs are very similar if not the same (e.g., Jenkins and Baird, 2002; Stamm, 2010) [9,39].

The factor analysis showed support for a three-factor solution for the scale. Except for two items, all the items fall into the original CBI factors [14], as found in the Iranian sample [28]. However, these two items are phrased such that participants may interpret them in various ways. These are the only items that were originally phrased in relation to work-related burnout and directly linked not to work, but to personal life related to work. For example, Slovak item number 7—CBI7 “Do you feel worn out at the end of the working day?”—has the slightly different meaning of feeling tired at the end of the whole day not just after work hours and so refers to the participant’s personal life and life as a whole. The situation is the same with item number 10—CBI10 “Do you have enough energy for family and friends during leisure time?” The Slovak translation implies that participants do not have enough energy for their personal life but not directly because they are exhausted from work. The problem with item 10 in the factor analysis has also been observed in a variety of studies (e.g., Campos et al., 2011; Yeh et al., 2007) e.g., [23,29], and the authors primarily interpreted it in terms of the reverse wording of the item. Nevertheless, in our sample there was no low factor loading of this item as it moved to a different factor. The authors of the CBI [14] did not employ factor analyses to validate the scale because they did not think that it was meaningful and thought it would not identify the three scales. However, they thought the three subscales were theoretically and methodologically justifiable. Our factor analysis supports the idea that the three subscales are theoretically and methodologically meaningful. This is despite the need to rephrase the two items, so they reflect the original factors of Kristensen et al. (2005) [14]. We suggest using the following wording, so these two items fit properly in work-related burnout:

CBI7 “Do you feel worn out at the end of the working day?” could be changed to “Do you feel worn out leaving your workplace?” and CBI10 “Do you have enough energy for family and friends during leisure time?” could be changed to “After work do you have enough energy for leisure time activities?”

### 8.1. Implications for Practice

The translated CBI and its analysis will enable further research on burnout in Slovakia, which is so important in the COVID-19 pandemic era, as the occurrence of burnout in some professions has doubled [40]. The Slovak version of the CBI could be used also for diagnostic purposes, which is highly needed due to impact of the COVID-19 pandemic, to prevent malpractice in many different areas of services. A sensitive instrument is necessary to reliably detect the level of burnout, as malpractice could yield excessive health expenditure and could hinder compliance with health treatment [41]. Apart from health services, it is important to assess burnout as it has adverse consequences in the form of mental and physical illnesses and economic losses.

### 8.2. Limitations

The second sample is asymmetrical in terms of gender as women account for more than 90% of the sample. The main limitation is that the sample is not representative of the Slovak population, and therefore, the results are not definitive.

### 8.3. Future Research

In future, we recommend providing statistical analyses of the factor structure, reliability, and validity for the rest of the scales relating to burnout in Slovakia as that would allow the use of more specifically tailored research instruments. In addition, we propose the validation of the CBI also via modern statistical techniques such as item response theory and structural equation modeling [42,43].

## 9. Conclusions

The Slovak version of the Copenhagen Burnout Inventory (CBI) [14] is a statistically sound instrument with high coefficients of reliability as well as validity and has an acceptable factor structure. The reliability coefficients varied between 0.839 and 0.879 for all subscales of the Slovak CBI. The validity analysis confirmed expected results of discriminant and convergent validity with related instruments Oldenburg Burnout Inventory (OLBI) [15], Professional Quality of Life Scale (PROQOL) [34], and Secondary Traumatic Stress Scale (STSS) [35]. The Slovak version of the CBI yielded a three factors structure with only two items, CBI10 and CBI17, loaded in a different factor than expected by their authors Kristensen et al. (2005) [14].

## Figures and Tables

**Table 1:** Descriptive analysis of the CBI items—sample one.

	Mean	Median	Interquartile Distance	Standard Deviation	Skewness		Kurtosis	
CBI Items					Statistic	Std. Error	Statistic	Std. Error
CBI1	2.71	3	1	0.675	−0.664	0.035	0.989	0.071
CBI2	2.38	2	1	0.779	−0.447	0.035	0.01	0.071
CBI3	2.51	3	1	0.829	−0.691	0.035	0.43	0.071
CBI4	2.05	2	2	0.952	−0.404	0.035	−0.446	0.071
CBI5	2.28	2	1	0.913	−0.51	0.035	−0.143	0.071
CBI6	1.82	2	1	0.949	−0.024	0.035	−0.537	0.071
CBI7	3.05	3	1	1.002	−0.986	0.035	0.182	0.071
CBI8	2.45	3	2	1.259	−0.363	0.035	−1.028	0.071
CBI9	1.93	2	2	1.25	0.128	0.035	−1.094	0.071
CBI10	1.93	2	1	0.89	−0.032	0.035	−0.053	0.071
CBI11	2.42	2	1	0.97	−0.401	0.035	−0.081	0.071
CBI12	2.02	2	2	0.996	−0.171	0.035	−0.274	0.071
CBI13	1.95	2	2	1.003	−0.213	0.035	−0.266	0.071
CBI14	1.52	1	1	0.97	0.861	0.041	−0.133	0.082
CBI15	2.23	2	2	1.091	−0.1	0.041	−1.043	0.082
CBI16	1.71	1	2	1.062	0.414	0.041	−0.612	0.082
CBI17	2.76	3	2	1.088	−0.708	0.041	−0.294	0.082
CBI18	2.44	3	2	1.12	−0.37	0.041	−0.847	0.082
CBI19	2.1	2	2	1.4	−0.043	0.041	−1.337	0.082
Personal burnout	13.7333			3.83555	−0.397	0.035	0.068	0.071
Work-related burnout	15.7371			5.33474	−0.251	0.035	−0.424	0.071
Client-related burnout	12.7689			5.09278	0.024	0.041	−0.564	0.082

Note. CBI = Copenhagen Burnout Inventory. OLBI = Oldenburg Burnout Inventory. PROQOL = Professional Quality of Life Scale. STSS = Secondary Traumatic Stress Scale.

**Table 2:** Descriptive analysis of the CBI items—sample two.

	Mean	Median	Interquartile Distance	Standard Deviation	Skewness		Kurtosis	
CBI Items					Statistic	Std. Error	Statistic	Std. Error
CBI1	2.44	2	1	0.982	−0.19	0.114	−0.44	0.228
CBI2	2.3	2	1	1.054	−0.132	0.114	−0.628	0.228
CBI3	2.24	2	2	1.15	−0.211	0.114	−0.856	0.228
CBI4	1.54	1	2	1.185	0.26	0.114	−1.01	0.228
CBI5	1.89	2	2	1.25	−0.007	0.114	−1.1	0.228
CBI6	1.6	2	1	1.157	0.301	0.114	−0.758	0.228
CBI7	2.86	3	2	1.182	−0.757	0.114	−0.457	0.228
CBI8	1.77	2	2	1.352	0.224	0.114	−1.104	0.228
CBI9	1.26	1	2	1.25	0.722	0.114	−0.502	0.228
CBI10	2.45	1	1	1.141	−0.363	0.114	−0.609	0.228
CBI11	2.79	3	2	1.126	−0.786	0.114	−0.071	0.228
CBI12	1.28	1	2	1.191	0.604	0.114	−0.663	0.228
CBI13	1.29	1	2	1.277	0.629	0.114	−0.744	0.228
CBI14	1.02	1	2	1.137	0.885	0.114	−0.184	0.228
CBI15	1.83	2	2	1.266	0.072	0.114	−1.014	0.228
CBI16	1.13	1	2	1.188	0.793	0.114	−0.324	0.228
CBI17	2.22	2	3	1.438	−0.237	0.114	−1.264	0.228
CBI18	1.94	2	2	1.256	0.047	0.114	−1.004	0.228
CBI19	1.84	2	2	1.404	0.081	0.114	−1.289	0.228
Personal burnout	12.0153			5.6295	0.072	0.114	−0.789	0.228
Work-related burnout	12.7991			6.38919	0.213	0.114	−0.713	0.228
Client-related burnout	9.9672			6.09166	0.257	0.114	−0.806	0.228

**Table 3:** Spearman correlations between the CBI subscales and the PROQOL, OLBI, and STSS subscales.

	Personal Burnout CBI	Work-Related Burnout CBI	Client-Related Burnout CBI
Compassion satisfaction PROQOL	−0.496 **	−0.544 **	−0.536 **
Burnout PROQOL	0.733 **	0.754 **	0.693 **
Compassion fatigue PROQOL	0.592 **	0.576 **	0.556 **
Disengagement OLBI	0.624 **	0.650 **	0.665 **
Exhaustion OLBI	0.779 **	0.798 **	0.730 **
Intrusion STSS	0.618 **	0.595 **	0.560 **
Avoidance STSS	0.743 **	0.737 **	0.718 **
Arousal STSS	0.750 **	0.716 **	0.687 **
Personal burnout CBI	1	0.855 **	0.790 **
Work-related burnout CBI	0.855 **	1	0.839 **
Client-related burnout CBI	0.790 **	0.839 **	1

Note. ** = *p* < 0.01.

**Table 4:** Pearson correlations between the CBI subscales and the PROQOL, OLBI, and STSS subscales.

	Personal Burnout CBI	Work-Related Burnout CBI	Client-Related Burnout CBI
Compassion satisfaction PROQOL	−0.505 **	−0.568 **	−0.568 **
Burnout PROQOL	0.729 **	0.761 **	0.707 **
Compassion fatigue PROQOL	0.596 **	0.581 **	0.580 **
Disengagement OLBI	0.602 **	0.636 **	0.659 **
Exhaustion OLBI	0.773 **	0.792 **	0.725 **
Intrusion STSS	0.638 **	0.604 **	0.585 **
Avoidance STSS	0.751 **	0.745 **	0.737 **
Arousal STSS	0.757 **	0.723 **	0.697 **
Personal burnout CBI	1	0.859 **	0.795 **
Work-related burnout CBI	0.859 **	1	0.843 **
Client-related burnout CBI	0.795 **	0.843 **	1

Note. ** = *p* < 0.01.

**Table 5:** Factor loadings of the CBI items.

CBI Items	Personal Burnout CBI	Work-Related Burnout CBI	Client-Related Burnout CBI	Commonalities
CBI1	**0.786**	0.106	0.008	0.537
CBI2	**0.780**	0.119	−0.001	0.512
CBI3	**0.499**	−0.207	0.015	0.424
CBI4	**0.516**	−0.322	−0.027	0.543
CBI5	**0.638**	−0.172	0.004	0.570
CBI6	**0.512**	−0.120	−0.003	0.346
CBI7	**0.581**	−0.049	0.089	0.434
CBI8	0.320	**−** **0.509**	0.041	0.593
CBI9	0.207	**−** **0.569**	0.082	0.582
CBI10	**0.523**	0.009	0.040	0.289
CBI11	0.060	**−** **0.364**	0.163	0.267
CBI12	−0.077	**−** **0.804**	0.089	0.666
CBI13	0.116	**−** **0.681**	0.081	0.651
CBI14	−0.044	0.007	**0.674**	0.423
CBI15	0.022	0.149	**0.878**	0.653
CBI16	−0.057	−0.106	**0.757**	0.634
CBI17	0.040	−0.070	**0.444**	0.260
CBI18	0.107	−0.005	**0.736**	0.630
CBI19	0.013	−0.208	**0.528**	0.461

Note. Bold = highest factor loading.

## Data Availability

In order to comply with the ethics approvals of the study protocols, data cannot be made accessible through a public repository. However, data are available upon request for researchers who consent to adhering to the ethical regulations for confidential data.

## References

[1] Freudenberger H.J. (1974). Staff burn-out. J. Soc. Issues.

[2] Maslach C. (1976). Burned-out. Hum. Behav..

[3] Brenninkmeijer V., Van Yperen N. (2003). How to conduct research on burnout: Advantages and disadvantages of a unidimensional approach in burnout research. Occup. Environ. Med..

[4] World Health Organization (2019). International Statistical Classification of Diseases and Related Health Problems.

[5] American Psychiatric Association (2013). Diagnostic and Statistical Manual of Mental Disorder.

[6] Bianchi R., Schonfeld I.S., Laurent E. (2015). Is it time to consider the “burnout syndrome” a distinct illness?. Front. Public Health.

[7] Thomas R.B., Wilson J.P. (2004). Issues and controversies in the understanding and diagnosis of compassion fatigue, vicarious traumatization, and secondary traumatic stress disorder. Int. J. Emerg. Ment. Health.

[8] Craig C.D., Sprang G. (2010). Compassion satisfaction, compassion fatigue, and burnout in a national sample of trauma treatment therapists. Anxiety Stress Coping.

[9] Stamm B.H. (2010). The Concise ProQOL Manual.

[10] Maslach C., Jackson S.E. (1981). The measurement of experienced burnout. J. Occup. Behav..

[11] Maslach C., Jackson S.E. (1986). Maslach Burnout Inventory Manual.

[12] Schaufeli W.B., Enzmann D. (1998). The Burnout Companion to Study and Practice: A Critical Analysis (p. 1998).

[13] Worley J.A., Vassar M., Wheeler D.L., Barnes L.L.B. (2008). Factor structure of scores from the Maslach Burnout Inventory: A review and meta-analysis of 45 exploratory and confirmatory factor-analytic studies. Educ. Psychol. Meas..

[14] Kristensen T.S., Borritz M., Villadsen E., Christensen K.B. (2005). The Copenhagen Burnout Inventory: A new tool for the assessment of burnout. Work Stress.

[15] Halbesleben J.R.B., Demerouti E. (2005). The construct validity of an alternative measure of burnout: Investigating the English translation of the Oldenburg Burnout Inventory. Work Stress.

[16] Salmela-Aro K., Kiuru N., Leskinen E., Nurmi J.-E. (2009). School Burnout Inventory: Reliability and validity. Eur. J. Psychol. Assess..

[17] Roskam I., Raes M.-E., Mikolajczak M. (2017). Exhausted parents: Development and preliminary validation of parental burnout inventory. Front. Psychol..

[18] Schaufeli W.B., De Witte H., Desart S. (2019). Burnout Assessment Tool (BAT)—Test Manual.

[19] Minihan E., Adamis D., Dunleavy M., Martin A., Gavin B., McNicholas F. (2021). COVID-19 related occupational stress in teachers in Ireland. Int. J. Educ. Res. Open.

[20] Papaefstathiou E., Tsounis A., Malliarou M., Sarafis P. (2019). Translation and validation of the Copenhagen Burnout Inventory amongst Greek doctors. Health Psychol. Res..

[21] Thrush C.R., Gathright M.M., Atkinson T., Messias E.L., Guise J.B. (2021). Psychometric properties of the Copenhagen Burnout Inventory in an academic healthcare institution sample in the US. Eval. Health Prof..

[22] Youssef D., Abou-Abbas L., Youssef J. (2022). Feeling the burn in the era of COVID-19: Cross-cultural adaptation and validation of the Arabic version of the Copenhagen Burnout inventory among community pharmacists. J. Pharm. Policy Pract..

[23] Yeh W.-Y., Cheng Y., Chen C.-J., Hu P.-Y., Kristensen T.S. (2007). Psychometric properties of the Chinese version of Copenhagen burnout inventory among employees in two companies in Taiwan. Int. J. Behav. Med..

[24] Fiorilli C., De Stasio S., Benevene P., Iezzi D.F., Pepe A., Albanese O. (2015). Copenhagen Burnout Inventory (CBI): A validation study in an Italian teacher group. TPM–Test. Psychom. Methodol. Appl. Psychol..

[25] Minamizono S., Nomura K., Inoue Y., Hiraike H., Tsuchiya A., Okinaga H. (2019). Gender division of labor, burnout, and intention to leave work among young female nurses in Japan: A cross-sectional study. Int. J. Environ. Res. Public Health.

[26] Jeon G.-S., You S.-J., Kim M.-G., Kim Y.-M., Cho S.-I. (2019). Psychometric properties of the Korean version of the Copenhagen burnout inventory in Korean homecare workers. PLoS ONE.

[27] Abdullah S., Ab Razak S., Yusoff M., Othman A., Yahaya N., Mohamad N. (2021). Burnout and stressor-related factors among caretaker of children with chronic neurological illness. Malays. J. Paediatr. Child Health.

[28] Mahmoudi S., Atashzadeh-Shoorideh F., Rassouli M., Moslemi A., Pishgooie A.H., Azimi H. (2017). Translation and psychometric properties of the Copenhagen burnout inventory in Iranian nurses. Iran. J. Nurs. Midwifery Res..

[29] Campos J., Zucoloto M.L., Bonafe F.S.S., Jordani P.C., Maroco J. (2011). Reliability and validity of self-reported burnout in college students: A cross randomized comparison of paper-and-pencil vs. online administration. Comput. Human Behav..

[30] Zotović M., Ukropina S., Mijatović-Jovanović V., Harhaji S. (2021). Burnout in healthcare professionals during COVID-19 pandemic: Correlates and predictors. Theme.

[31] Gjura A. (2021). Izgorelost Zdravstvenih Delavcev v Socialno Varstvenem Zavodu Med Epidemijo COVID-19. https://dk.um.si/IzpisGradiva.php?id=80761.

[32] Castro A. (2021). Assessment of Burnout in Internal Medicine and Psychiatry at the Doce de Octubre Hospital in Madrid using the Copenhagen Burnout Inventory: A Cross-sectional Observational Study. EIDON.

[33] Wongtrakul W., Dangprapai Y., Saisavoey N., Sa-nguanpanich N. (2021). Reliability and validity study of the Thai adaptation of the Copenhagen Burnout Inventory-Student Survey (CBI-SS) among preclinical medical students at the Faculty of Medicine Siriraj Hospital, Mahidol University, Thailand. PLoS ONE.

[34] Stamm B.H. (2009). Professional Quality of Life: Compassion Satisfaction and Fatigue Version 5 (ProQOL).

[35] Bride B.E., Robinson M.M., Yegidis B., Figley C.R. (2004). Development and validation of the Secondary Traumatic Stress Scale. Res. Soc. Work. Pract..

[36] Ondrejková N., Halamová J. (2022). The psychometric properties and factor analysis of slovak versions of The Oldenburg Burnout Inventory and The Secondary Traumatic Stress Scale.

[37] Köverová M. (2016). Psychometric properties of the Slovak version of the professional quality of life scale: Preliminary results. Glob. J. Psychol. Res. New Trends Issues.

[38] Köverová M. (2018). Konfirmačná faktorová analýza slovenskej verzie škály profesijnej kvality života (ProQOL). Ceskoslovenska Psychol..

[39] Jenkins S.R., Baird S. (2002). Secondary traumatic stress and vicarious trauma: A validational study. J. Trauma. Stress.

[40] Gewin V. (2021). Pandemic burnout is rampant in academia. Nature.

[41] Liang F., Hu S., Guo Y. (2022). The association between fear of malpractice and burnout among Chinese medical workers: The mediating role of legal consciousness. BMC Psychiatry.

[42] Barbosa A.S., da Silva L.B., Morioka S.N., da Silva J.M.N., de Souza V.F. (2021). Item response theory-based validation of an integrated management system measurement instrument. J. Clean. Prod..

[43] Souza D.S.F.D., Silva J.M.N.D., Santos J.V.D.O., Alcantara M.S.B., Torres M.G.L. (2021). Influence of risk factors associated with musculoskeletal disorders on an inner population of northeastern Brazil. Int. J. Ind. Ergon..

